# Supplementary material for: Hemodynamic performance and blood damage of the Intra-aortic pumps: A CFD-Based investigation
Source: Med Biol Eng Comput. 2026 Mar 21;64(5):1853–69. doi: 10.1007/s11517-026-03550-y (PMC13133241; doi:10.1007/s11517-026-03550-y)
Supplement: Supplementary file 1 — (DOCX 6.83 MB) [file 11517_2026_3550_MOESM1_ESM.docx]

**Hemodynamic Performance and Blood Damage of the Intra-Aortic Pumps: A CFD-Based Investigation**

**Osman Aycan^1,2^, Yeojin Park^1^, Lyes Kadem^1^**

^1^Laboratory of Cardiovascular Fluid Dynamics, Department of Mechanical Industrial and Aerospace Engineering, Concordia University, Montreal, QC, Canada

^2^Department of Mechanical Engineering, Faculty of Engineering, Zonguldak Bulent Ecevit University, Zonguldak, Türkiye

**Correspondence**

Osman Aycan, Department of Mechanical Engineering, Faculty of Engineering, Zonguldak Bulent Ecevit University, Zonguldak, Türkiye.

Email: [osman.aycan@beun.edu.tr](mailto:osman.aycan@beun.edu.tr); [lcfd@encs.concordia.ca](mailto:lcfd@encs.concordia.ca). Tel: +90 (372) 291 1144

*Supplementary Material*

**A. Grid Study with Grid Convergence Index (GCI) Method**

In this study, the evaluation of numerical uncertainties was combined with grid independence study. For each pump geometry, five distinct grid levels from coarse (case 5) to fine (case 1) were generated for the single, triplet and impeller-driven pump, as detailed in **Table A1**, **Table A2** and **Table A3**, respectively. Minimum orthogonal quality and maximum aspect ratio were considered as quality measures of the solution grids.

**Table A1** Overview of the generated mesh for the impeller-driven pump^*^

|  | Cell Sizes  [Surface Mesh] | | | Cell Sizes  [Volume Mesh] | | Near-Wall  Boundary Layers | | Cell Count | Quality Measure^**^ | | Flow Rate |
| --- | --- | --- | --- | --- | --- | --- | --- | --- | --- | --- | --- |
|  | ***Pump Blades***  ***(Min*** $\boldsymbol{\sim}$ ***Max)*** | ***Main Body***  ***and Motor***  ***(Min*** $\boldsymbol{\sim}$ ***Max)*** | ***Pipe Wall*** | ***Rotating Domain***  ***(Min*** $\boldsymbol{\sim}$ ***Max)*** | ***Pipe***  ***(Min*** $\boldsymbol{\sim}$ ***Max)*** | ***Pipe***  ***Wall*** | ***Main***  ***Body*** | ***Polyhedral and Prism Elements*** | ***Minimum Orthogonal*** | ***Max. Aspect Ratio*** | ***Difference***  ***(%)*** |
| Case 5 | 0.060 $\boldsymbol{\sim}$ 0.30 | 0.060 $\boldsymbol{\sim}$ 0.30 | 1.20 | 0.06 $\boldsymbol{\sim}$ 0.6 | 0.3 $\boldsymbol{\sim}$ 1.20 | 9 | 3 | 534,890 | 0.294 | 25.0 | - |
| Case 4 | 0.046 $\boldsymbol{\sim}$ 0.23 | 0.046 $\boldsymbol{\sim}$ 0.23 | 0.92 | 0.046 $\boldsymbol{\sim}$ 0.46 | 0.23 $\boldsymbol{\sim}$ 0.92 | 9 | 3 | 822,825 | 0.314 | 19.3 | 1.15 |
| Case 3 | **0.035** $\boldsymbol{\sim}$ **0.18** | **0.035** $\boldsymbol{\sim}$ **0.18** | **0.71** | **0.035** $\boldsymbol{\sim}$ **0.35** | **0.18** $\boldsymbol{\sim}$ **0.71** | **9** | **3** | **1,148,563** | **0.325** | **14.5** | **0.94** |
| Case 2 | 0.027 $\sim$ 0.14 | 0.027 $\boldsymbol{\sim}$ 0.14 | 0.55 | 0.027 $\boldsymbol{\sim}$ 0.27 | 0.14 $\sim$ 0.55 | 9 | 3 | 1,906,818 | 0.329 | 11.1 | 0.71 |
| Case 1 | 0.021 $\sim$ 0.11 | 0.021 $\boldsymbol{\sim}$ 0.11 | 0.42 | 0.021 $\boldsymbol{\sim}$ 0.21 | 0.11 $\sim$ 0.42 | 9 | 3 | 3,563,604 | 0.333 | 8.72 | 0.32 |
| * All dimensions are given in millimeters (mm) | | | | | | **** more than 99.99% of all elements number** | | | | | |

**Table A2** Overview of the generated mesh for the single pump^*^

|  | Cell Sizes  [Surface Mesh] | | | Cell Sizes  [Volume Mesh] | | Local Refinement | Near-Wall Boundary Layers | | Cell Count | Quality Measure^**^ | | Flow Rate |
| --- | --- | --- | --- | --- | --- | --- | --- | --- | --- | --- | --- | --- |
|  | ***Pump***  ***(Min*** $\boldsymbol{\sim}$ ***Max)*** | ***Casing and Motor***  ***(Min*** $\boldsymbol{\sim}$ ***Max)*** | ***Pipe Wall*** | ***Pump***  ***(Min*** $\boldsymbol{\sim}$ ***Max)*** | ***Pipe***  ***(Min*** $\boldsymbol{\sim}$ ***Max)*** | ***Entrance Zone to Pumps in Pipe*** | ***Pipe Wall*** | ***Casing and Motor*** | ***Polyhedral and Prism Elements*** | ***Minimum Orthogonal*** | ***Max. Aspect Ratio*** | ***Difference***  ***(%)*** |
| Case 5 | 0.060 $\boldsymbol{\sim}$ 0.30 | 0.10 $\boldsymbol{\sim}$ 0.50 | 1.5 | 0.06 $\boldsymbol{\sim}$ 0.60 | 0.30 $\boldsymbol{\sim}$ 1.50 | 0.30 | 9 | 5 | 545,037 | 0.293 | 29.0 | - |
| Case 4 | 0.046 $\boldsymbol{\sim}$ 0.23 | 0.77 $\boldsymbol{\sim}$ 0.38 | 1.15 | 0.046 $\boldsymbol{\sim}$ 0.46 | 0.23 $\sim$ 1.15 | 0.23 | 9 | 5 | 734,639 | 0.268 | 21.4 | 2.74 |
| Case 3 | **0.035** $\boldsymbol{\sim}$ **0.18** | **0.06** $\boldsymbol{\sim}$ **0.30** | **0.88** | **0.035** $\boldsymbol{\sim}$ **0.35** | **0.18** $\boldsymbol{\sim}$ **0.88** | **0.18** | **9** | **5** | **1,212,130** | **0.266** | **17.0** | **1.79** |
| Case 2 | 0.027 $\sim$ 0.14 | 0.045 $\boldsymbol{\sim}$ 0.23 | 0.67 | 0.027 $\boldsymbol{\sim}$ 0.27 | 0.14 $\sim$ 0.67 | 0.14 | 9 | 5 | 2,031,614 | 0.232 | 12.8 | 1.65 |
| Case 1 | 0.021 $\sim$ 0.11 | 0.035 $\boldsymbol{\sim}$ 0.18 | 0.52 | 0.021 $\boldsymbol{\sim}$ 0.21 | 0.11 $\sim$ 0.52 | 0.11 | 9 | 5 | 3,526,742 | 0.219 | 9.95 | 0.11 |
| * All dimensions are given in millimeters (mm) | | | | | | | **** more than 99.99% of all elements number** | | | | | |

**Table A3** Overview of the generated mesh for the triplet pump^*^

|  | Cell Sizes  [Surface Mesh] | | | Cell Sizes  [Volume Mesh] | | Local Refinement | Near-Wall  Boundary Layers | | Cell Count | Quality Measure^**^ | | Flow Rate |
| --- | --- | --- | --- | --- | --- | --- | --- | --- | --- | --- | --- | --- |
|  | ***Pump***  ***(Min*** $\boldsymbol{\sim}$ ***Max)*** | ***Casing and Motor***  ***(Min*** $\boldsymbol{\sim}$ ***Max)*** | ***Pipe Wall*** | ***Pump***  ***(Min*** $\boldsymbol{\sim}$ ***Max)*** | ***Pipe***  ***(Min*** $\boldsymbol{\sim}$ ***Max)*** | ***Entrance Zone to Pumps in Pipe*** | ***Pipe Wall*** | ***Casing and Motor*** | ***Polyhedral and Prism Elements*** | ***Minimum Orthogonal*** | ***Max. Aspect Ratio*** | ***Difference***  ***(%)*** |
| Case 5 | 0.04 $\boldsymbol{\sim}$ 0.20 | 0.06 $\boldsymbol{\sim}$ 0.30 | 1.5 | 0.04 $\boldsymbol{\sim}$ 0.40 | 0.30 $\boldsymbol{\sim}$ 1.50 | 0.20 | 9 | 5 | 820,504 | 0.28 | 30.2 | - |
| Case 4 | 0.031 $\boldsymbol{\sim}$ 0.15 | 0.046 $\boldsymbol{\sim}$ 0.23 | 1.15 | 0.031 $\boldsymbol{\sim}$ 0.31 | 0.23 $\boldsymbol{\sim}$ 1.15 | 0.15 | 9 | 5 | 1,271,794 | 0.29 | 22.7 | 3.16 |
| Case 3 | **0.024** $\boldsymbol{\sim}$ **0.12** | **0.035** $\boldsymbol{\sim}$ **0.18** | **0.88** | **0.024** $\boldsymbol{\sim}$ **0.24** | **0.18** $\boldsymbol{\sim}$ **0.88** | **0.12** | **9** | **5** | **1,951,386** | **0.30** | **17.4** | **1.25** |
| Case 2 | 0.018 $\boldsymbol{\sim}$ 0.09 | 0.027 $\boldsymbol{\sim}$ 0.14 | 0.67 | 0.018 $\boldsymbol{\sim}$ 0.18 | 0.14 $\boldsymbol{\sim}$ 0.67 | 0.09 | 9 | 5 | 3,184,512 | 0.30 | 12.9 | 0.72 |
| Case 1 | 0.014 $\boldsymbol{\sim}$ 0.07 | 0.021 $\boldsymbol{\sim}$ 0.11 | 0.52 | 0.014 $\boldsymbol{\sim}$ 0.14 | 0.11 $\boldsymbol{\sim}$ 0.52 | 0.07 | 9 | 5 | 5,378,273 | 0.31 | 10.2 | 0.41 |
| * All dimensions are given in millimeters (mm) | | | | | | | **** more than 99.99% of all elements number** | | | | | |

| **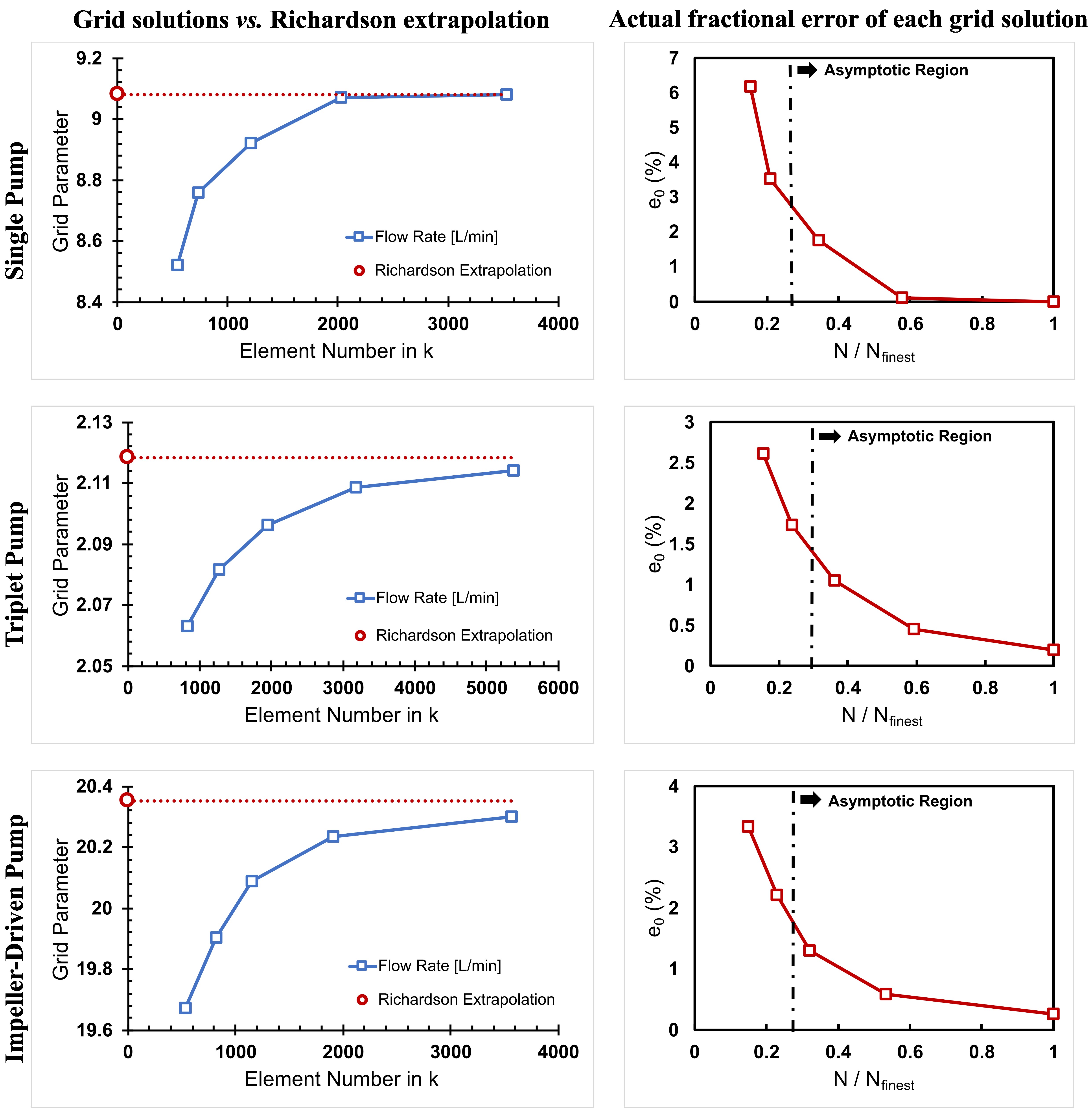** |
| --- |
| **Figure A1** The comparison of the grid solutions and Richardson extrapolation with actual fractional errors |

The GCI method [1] is based on generalized Richardson extrapolation [2], and it compares the results of different mesh sizes (from case 5 to case 1). The grid refinement ratio between successive meshes is defined as $r={h_{k}}/{h_{j}}={h_{j}}/{h_{i}}=1.3$, where $h$ is a representative grid size, and $i$, $j$, $k$ represent fine, medium, coarse grid resolutions, respectively. The order of convergence of the solution can be obtained with,

| $p=\left\vert\ln\left( \frac{f_{k}-f_{j}}{f_{j}-f_{i}} \right) \right\vert/\ln(r)$ | (A.1) |
| --- | --- |

where $f_{i}$, $f_{j}$ and $f_{k}$ are grid solution values on fine, medium and coarse grids. The discretization error is used to calculate an error band, and this defines for the fine grid solution as,

| ${GCI}_{ij}=F_{s}\cdot\left\vert\frac{f_{j}-f_{i}}{f_{i}} \right\vert\cdot\frac{1}{r^{p}-1}$ | (A.2) |
| --- | --- |

where $F_{s}$ is a safety factor, and it is used as 1.25 due to three or more grid spacings. The continuum value at the zero-grid spacing $f_{h=0}$ (Richardson Extrapolation) replaces the analytical solution $f_{exact}$, and it is calculated as,

| $f_{h=0}=f_{i}+\frac{f_{i}-f_{j}}{r^{p}-1}$ | (A.3) |
| --- | --- |

According to results of mesh independence analysis using the GCI method, the actual fractional error $e_{0} (\%)$ was calculated for each mesh case of every pump geometry using Richardson extrapolation. The comparison of the grid solutions and Richardson extrapolation with actual fractional errors is given in **Figure A1**. The value of $f_{h=0}$ was calculated as 9.08 L/min, 2.12 L/min, and 20.35 L/min for single, triplet, and impeller-driven pumps, respectively. These grid solutions also represent the maximum capacity for generating flow under the specified simulation conditions for each pump device.

The flow rate difference between two different mesh cases is under 5% for each pump configuration, with the maximum difference of 3.16% occurring between Case 5 and Case 4 for the triplet-pump as shown in **Table A1, A2 and A3**. However, uncertainties should be considered when evaluating and specifying the optimum grid size. The actual fractional error of the grid solutions exceeds 5% for the single and triplet-pump with coarsest grid size, while it is approximately 3.5% for impeller-driven.

**Table A4** Uncertainties between each mesh case of all pump geometries

|  |  | Single Pump | | Triplet Pump | | Impeller-driven Pump | |
| --- | --- | --- | --- | --- | --- | --- | --- |
| Element Type | **Mesh Cases** | **GCI Value (*%*)** | | **GCI Value (*%*)** | | **GCI Value (*%*)** | |
|  | **(*i j k*)** | $\boldsymbol{GCI}_{\boldsymbol{ij}}$ | $\boldsymbol{GCI}_{\boldsymbol{jk}}$ | $\boldsymbol{GCI}_{\boldsymbol{ij}}$ | $\boldsymbol{GCI}_{\boldsymbol{jk}}$ | $\boldsymbol{GCI}_{\boldsymbol{ij}}$ | $\boldsymbol{GCI}_{\boldsymbol{jk}}$ |
| Polyhedral | 1 2 3 | 0.01 | 0.14 | 0.24 | 0.57 | 0.33 | 0.74 |
|  | 2 3 4 | 3.10 | 3.36 | 5.05 | 5.84 | 2.89 | 3.81 |
|  | 3 4 5 | 4.48 | 6.85 | 3.07 | 3.96 | 5.39 | 6.62 |

In this grid study, uncertainty and error bands were set to a maximum 2%, ensuring that the hemodynamic and hemolytic performance of the pumps could be evaluated with high accuracy using wall-modeled LES. From this perspective, the mesh cases for each pump geometry that fall below the 2% error threshold and lie within the asymptotic region are shown in the right column of **Figure A1**, based on the GCI values provided in **Table A4**. The GCI values are under 2% for the triplet mesh group (Case 1-2-3) of every pump geometry. Moreover, the triplet mesh group (Case 1-2-3) demonstrates *good monotonic convergence* condition for all pumps.

| 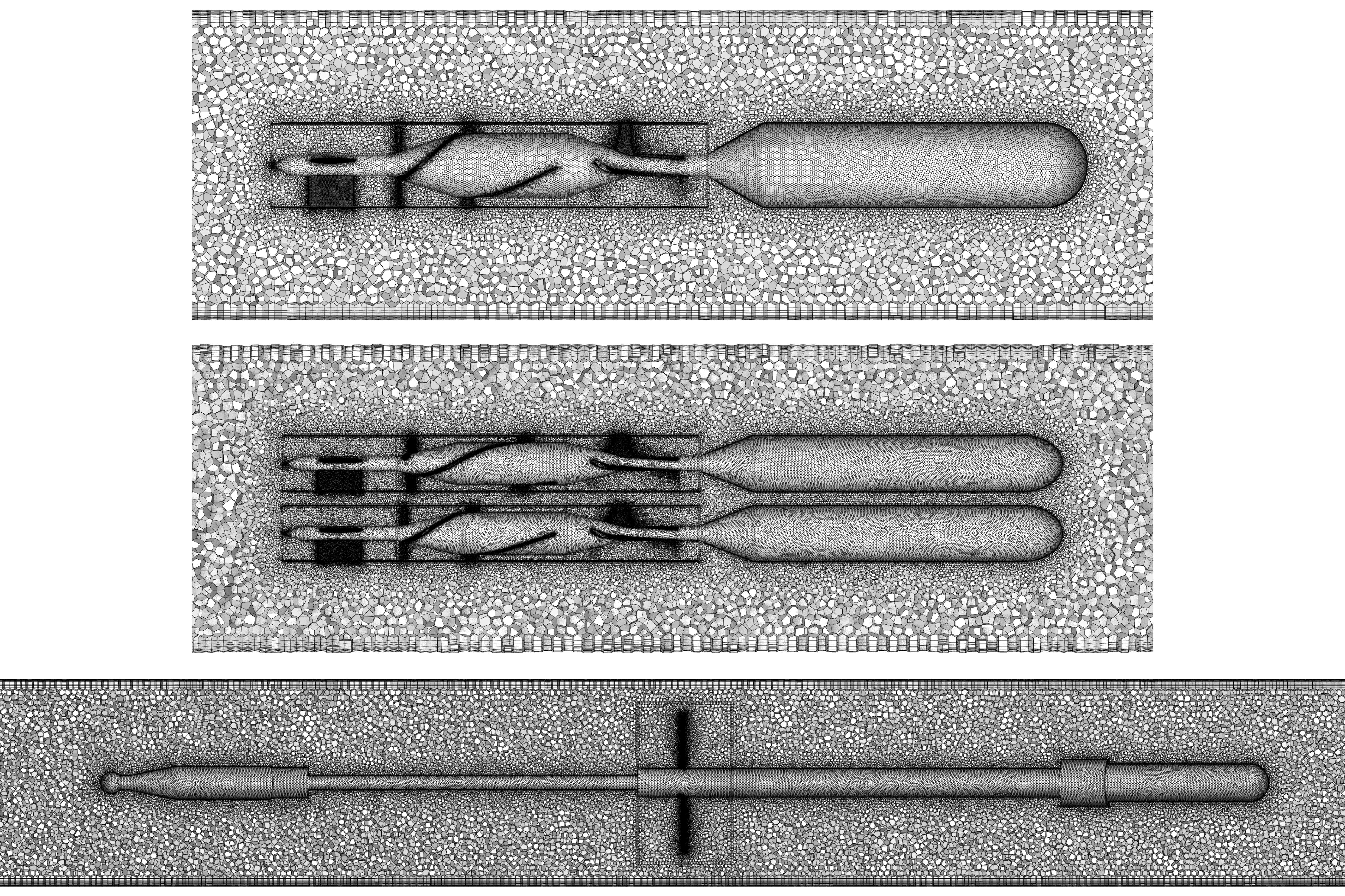 |
| --- |
| **Figure A2** Mesh details of the studied pumps; *top:* single pump, *middle:* triplet-pump, and *bottom:* impeller-driven pump |

As a result of this analysis, the selected mesh cases are Case 3 with 1.21 million element number for single pump, Case 3 with 1.95 million element number for triplet-pump, and Case 3 with 1.15 million element number for impeller-driven pump. The view of selected mesh cases is shown in **Figure A2** for each pump type.

**B. Governing Equations of LES**

The governing equations of LES are obtained by applying a spatial filter to incompressible Navier-Stokes (N-S) equations as follows [3, 4]:

| $\frac{\partial\tilde{u}_{i}}{\partial x_{i}}=0$ | (B.1) |
| --- | --- |

| $\frac{\partial\tilde{u}_{i}}{\partial t}+\frac{\partial\tilde{u}_{i}\tilde{u}_{j}}{\partial x_{j}}=-\frac{1}{\rho}\frac{\partial\tilde{p}}{\partial x_{i}}+\upsilon\frac{\partial^{2}\tilde{u}_{i}}{\partial x_{j}x_{j}}+\frac{\partial{\tau_{ij}}^{SGS}}{\partial\tilde{x}_{j}}$ | (B.2) |
| --- | --- |

where $\tilde{u}_{i}$ is the filtered velocity component, $\tilde{p}$ is the filtered pressure, $\rho$ is the density, $\upsilon$ is the kinematic viscosity, and ${\tau_{ij}}^{SGS}$ is the subgrid-scale (SGS) stress tensor. A new term, the SGS stress tensor (${\tau_{ij}}^{SGS}$), was added to the classical N-S equations.

The SGS tensor is modeled using the Smagorinsky definition based on the balance between the SGS energy production and dissipation as follows [5]:

| ${\tau_{ij}}^{SGS}=\frac{2}{3}k_{SGS}\delta_{ij}-2\upsilon_{SGS}\tilde{S}_{ij}$ | (B.3) |
| --- | --- |

where $\upsilon_{SGS}$ is the SGS eddy viscosity, and it defines as $\upsilon_{SGS}=C_{k}\Delta\sqrt{k_{SGS}}$ with $C_{k}=0.094$. The rate of stress tensor $\tilde{S}_{ij}$ is also calculated using filtered velocity components,

| $\tilde{S}_{ij}=\frac{1}{2}\left( \frac{\partial\tilde{u}_{i}}{\partial x_{j}}+\frac{\partial\tilde{u}_{j}}{\partial x_{i}} \right)$ | (B.4) |
| --- | --- |

and the eddy viscosity $\upsilon_{SGS}$ is computed by one of the popular SGS models, such as the Smagorinsky model, the dynamic Smagorinsky model, and the Wall-Adapting Local Eddy viscosity (WALE) model, etc.

For the wall modeled LES, the wall stress is characterized in the inner boundary layer within the thin boundary layer equations [6, 7],

| $\frac{d}{dy}\left[ (\mu+\mu_{t})\frac{du}{dy} \right]=0$ | (B.5) |
| --- | --- |

where the turbulent eddy viscosity can be defined as $\mu_{t}=\kappa\mu y^{+}{[1-exp(-y^{+}/A^{+})]}^{2}$, and $y^{+}=yu_{\tau}/v$ is the normalized distance to the wall with the kinematic viscosity.

**C. Dimensionless Number**

The derivation and clarification of HN have been substantially expanded, as shown in Table C1.

**Table C1.** Detailed description of the steps that comprise the method of repeating variables to derive HN

| **Step 1** | | |
| --- | --- | --- |
| There are five parameters that affect the blood damage analysis in this problem. | | |
| *List of relevant parameters:* | $\tau=f(t_{exp},\rho,\omega,D)$ | $n=5$ |

| **Step 2** | | | | |
| --- | --- | --- | --- | --- |
| The primary dimensions of each parameter are listed here. | | | | |
| $\tau$ | $t_{exp}$ | $\rho$ | $\omega$ | $D$ |
| $\left\{ mL^{-1}t^{-2} \right\}$ | $\left\{ t \right\}$ | $\left\{ mL^{-3} \right\}$ | $\left\{ t^{-1} \right\}$ | $\left\{ L \right\}$ |

| **Step 3** | | |
| --- | --- | --- |
| The problem is characterized by the presence of three primary dimensions ($m,L,T$). | | |
| *Reduction:* | $j=3$ | |
| The number of $\Pi$’s is predicted by the Buckingham Pi theorem. | | |
| *Number of expected* $\Pi$*’s:* | | $k=n-j=5-3=2$ |

| **Step 4** | |
| --- | --- |
| We need to choose three repeating parameters since $j=3$. | |
| *Repeating parameters:* | $\rho$, $\omega$ and $D$ |

| **Step 5** | | | | |
| --- | --- | --- | --- | --- |
| Now let's group the $\Pi$ 's by multiplying these repeating parameters individually with each of the remaining ones. | | | | |
| *Dependent* $\Pi$*:* | | $\prod_{1} =\tau\cdot\rho^{a_{1}}\cdot\omega^{b_{1}}\cdot D^{c_{1}}$ | | |
| where $a_{1}$, $b_{1}$ and $c_{1}$ are constant exponents that need to be determined. | | | | |
| *Dimensions of* $\Pi_{1}$*:* | | | | |
| $\left\{ \Pi_{1} \right\}=\left\{ m^{0}L^{0}t^{0} \right\}=\left\{ \tau\cdot\rho^{a_{1}}\cdot\omega^{b_{1}}\cdot D^{c_{1}} \right\}=\{(mL^{-1}t^{-2})\cdot{(mL^{-3})}^{a_{1}}\cdot{(t^{-1})}^{b_{1}}\cdot{(L)}^{c_{1}}\}$ | | | | |
| Mass | : ${\{m}^{0}\}={\{m}^{1+a_{1}}\}$ | | $0=1+a_{1}$ | $a_{1}=-1$ |
| Length | : ${\{L}^{0}\}={\{L}^{-1-3a_{1}+c_{1}}\}$ | | $0=-1-3a_{1}+c_{1}$ | $c_{1}=-2$ |
| Time | : ${\{t}^{0}\}={\{t}^{-2-b_{1}}\}$ | | $0=-2-b_{1}$ | $b_{1}=-2$ |
| $\prod_{1} =\tau\cdot\rho^{-1}\cdot\omega^{-2}\cdot D^{-2}=\frac{\tau}{\rho\cdot\omega^{2}\cdot D^{2}}$ | | | | |
| *Independent* $\Pi$*:* | | $\prod_{2} =t_{exp}\cdot\rho^{a_{2}}\cdot\omega^{b_{2}}\cdot D^{c_{2}}$ | | |
| *Dimensions of* $\Pi_{2}$*:* | | | | |
| $\left\{ \Pi_{2} \right\}=\left\{ m^{0}L^{0}t^{0} \right\}=\left\{ t_{exp}\cdot\rho^{a_{2}}\cdot\omega^{b_{2}}\cdot D^{c_{2}} \right\}=\{(t)\cdot{(mL^{-3})}^{a_{2}}\cdot{(t^{-1})}^{b_{2}}\cdot{(L)}^{c_{2}}\}$ | | | | |
| Mass | : ${\{m}^{0}\}={\{m}^{a_{2}}\}$ | | $0=a_{2}$ | $a_{2}=0$ |
| Length | : ${\{L}^{0}\}={\{L}^{-3a_{2}+c_{2}}\}$ | | $0=-3a_{2}+c_{2}$ | $c_{2}=0$ |
| Time | : ${\{t}^{0}\}={\{t}^{1-b_{2}}\}$ | | $0=1-b_{2}$ | $b_{2}=1$ |
| $\prod_{2} =t_{exp}\cdot\rho^{a_{2}}\cdot\omega^{b_{2}}\cdot D^{c_{2}}=t_{exp}\cdot\omega$ | | | | |

| **Step 6** | |
| --- | --- |
| Check that all the $\Pi$’s are indeed dimensionless. | |
| *Relationship between* $\Pi$*’s:* | $f\left( \Pi_{1},\Pi_{2} \right)=0$ |
| The Hemolytic Number (HN) is defined as a combined dimensionless measure following rearrangement. | |
| $HN=\Pi_{1}\cdot\Pi_{2}=\frac{\tau}{\rho\cdot\omega^{2}\cdot D^{2}}\cdot t_{exp}\cdot\omega=\frac{\tau\cdot t_{exp}}{\rho\cdot\omega\cdot D^{2}}$ | |

**References**

1. Roache PJ (1994) Perspective: A Method for Uniform Reporting of Grid Refinement Studies. J Fluids Eng 116:405–413

2. Richardson LF (1911) IX. The approximate arithmetical solution by finite differences of physical problems involving differential equations, with an application to the stresses in a masonry dam. Philosophical Transactions of the Royal Society of London Series A, Containing Papers of a Mathematical or Physical Character 210:307–357

3. Shi Y, Kollmann W (2021) Wall-modeled large-eddy simulation of a trailing-edge serration-finlet configuration. AIP Adv 11:. https://doi.org/10.1063/5.0049181

4. Ren X, Su H, Yu HH, Yan Z (2022) Wall-Modeled Large Eddy Simulation and Detached Eddy Simulation of Wall-Mounted Separated Flow via OpenFOAM. Aerospace 9:. https://doi.org/10.3390/aerospace9120759

5. Smagorinsky J (1963) General Circulation Experiments with the Primitive Equations: I. The Basic Experiment. Mon Weather Rev 91:99–164

6. Larsson J, Kawai S, Bodart J, Bermejo-Moreno I (2016) Large eddy simulation with modeled wall-stress: recent progress and future directions. Mechanical Engineering Reviews 3:1–23. https://doi.org/10.1299/mer.15-00418

7. Bose ST, Park GI (2018) Wall-Modeled Large-Eddy Simulation for Complex Turbulent Flows. Annu Rev Fluid Mech 50:535–561. https://doi.org/10.1146/annurev-fluid-122316
